# Evidence from Human Studies for Utilising Cannabinoids for the Treatment of Substance-Use Disorders: A Scoping Review with a Systematic Approach

**DOI:** 10.3390/ijerph20054087

**Published:** 2023-02-24

**Authors:** Kayvan Ali Gharbi, Yvonne Ann Bonomo, Christine Mary Hallinan

**Affiliations:** 1 Department of General Practice, Faculty of Medicine, Dentistry and Health Sciences, The University of Melbourne, Parkville, VIC 3010, Australia; 2 Department of Medicine, Faculty of Medicine, Dentistry and Health Sciences, The University of Melbourne, Parkville, VIC 3010, Australia; 3 St Vincent’s Health—Department of Addiction Medicine, Fitzroy, VIC 3065, Australia; 4 Health & Biomedical Research Information Technology Unit (HaBIC R2), Department of General Practice, Melbourne Medical School, Faculty of Medicine, Dentistry and Health Sciences, The University of Melbourne, Parkville, VIC 3010, Australia

**Keywords:** substance-use disorders, cannabinoids, dronabinol, cannabidiol, treatment

## Abstract

Substance-use disorders are pervasive, comorbid with a plethora of disease and possess limited treatment options. Medicinal cannabinoids have been proposed as a novel potential treatment based on preclinical/animal trials. The objective of this study was to examine the efficacy and safety of potential therapeutics targeting the endocannabinoid system in the treatment of substance-use disorders. We performed a scoping review using a systematic approach of systematic reviews, narrative reviews, and randomised control trials that utilised cannabinoids as treatment for substance-use disorders. For this scoping review we used the PRISMA guidelines, a framework for systematic reviews and meta-analyses, to inform our methodology. We conducted a manual search of Medline, Embase, and Scopus databases in July 2022. Of the 253 results returned by the databases, 25 studies including reviews were identified as relevant, from which 29 randomised controlled trials were derived and analysed via a primary study decomposition. This review captured a small volume of highly heterogenous primary literature investing the therapeutic effect of cannabinoids for substance-use disorders. The most promising findings appeared to be for cannabis-use disorder. Cannabidiol appeared to be the cannabinoid showing the most promise for the treatment of multiple-substance-use disorders.

## 1. Introduction

### 1.1. Substance-Use Disorders

Substance-use disorders are prevalent across Australia, with 3.3% of individuals aged between 16 and 85 years possessing a substance-use disorder of at least 12 months, with alcohol the principal substance of abuse at 2.5% [1]. Substance-use disorders are comorbid with both psychiatric (mood, psychotic, trauma, anxiety) [2] and physical disease (HIV, STIs, CVD, chronic pain, opportunistic infection) [3]. Mental health and substance-use disorders comprise 13% of Australia’s total burden of disease, making them the fourth most significant disease group [4]. Treatment options for substance-use disorders remain relatively limited, though there is strong evidence for medications (particularly agonist therapies) such as methadone and buprenorphine for opioid-use disorder [5], nicotine-replacement therapy for smoking cessation [6], and ‘anti-craving’ medications for alcohol-use disorder [7].The effectiveness of psychosocial interventions for substance-use disorders is mixed across the literature, with patient reluctance being an important contributor to the low utilisation of treatments considered effective [8]. Given the prevalence of substance-use disorders and their limited treatment options, efficacious therapeutics are needed. Medicinal cannabinoids have been proposed as a potential treatment option [9].

### 1.2. The Endocannabinoid System

The endocannabinoid system is a complex system of receptors, their ligands (endocannabinoids), and regulatory enzymes. There are two types of cannabinoid receptor (CB1R, CB2R) and two primary endocannabinoids: anandamide (ANA), also known as arachidonoylethanolamide (AEA), and 2-arachidonoylglycerol (2-AG). Each endocannabinoid is degraded by the enzymes fatty-acid amide hydrolase (FAAH) and monoacylglycerol lipase (MAGL), respectively [10].

Preclinical studies of substance-use disorder treatments have applied both organic and synthetic molecules within the endocannabinoid system. Those at the stage of human trials include CB1 receptor agonists (tetrahydrocannabinol, dronabinol, nabiximols), receptor modulators (cannabidiol), enzyme inhibitors (FAAH Inhibitors), CB1 receptor inverse agonists (rimonabant, taranabant), and CB1 receptor antagonists (surinabant). Tetrahydrocannabinol (THC) is a naturally occurring exogenous cannabinoid that is orally bioavailable and the chief psychoactive component of cannabis acting at the CB1 receptor. Dronabinol is a synthetic enantiomer of THC. Cannabidiol (CBD) is an alternative cannabinoid with minimal direct action at receptors; nonetheless, it possesses a wide array of effects including allosteric modification of both endocannabinoid receptors [11] and opioid receptors [12], whilst inhibiting the hydrolysis and reuptake of AEA (increasing the availability of this CB1 receptor agonist) [13]. Nabiximols are a whole-plant-extract combined formulation of THC and CBD. They are typically delivered at an even dosage ratio in the form of an oromucosal spray that engenders a more predictable pharmacokinetic profile than oral dronabinol [14]. FAAH (fatty-acid amide hydrolase) inhibitors augment CB1 activation via increasing concentrations of the endocannabinoid AEA through inhibition of the enzyme FAAH thereby inhibiting degradation of AEA. CB1 inverse agonists and antagonists inhibit the downstream effects of endocannabinoids upon dopaminergic release in the nucleus accumbens [15], a mechanism central to the development of substance dependence [16].

Given growing preclinical evidence for several differing mechanisms of action of medicinal cannabinoids in the neurobiological pathways of substance-use disorders, findings from human studies were of interest. Hence, the objective of this scoping review is to synthesise primary research investigating the effect of cannabinoids on substance-use disorders to answer the following question: What is the efficacy and safety of potential therapeutics targeting the endocannabinoid system in the treatment of substance-use disorders in humans?

## 2. Materials and Methods

The review questions were decomposed using the PICOS search algorithm [17] to guide the development of search terms for the systematic review of reviews. The PICOS acronym refers to: P–participants; I–intervention; C–control; O–outcome; and S–study design (Table 1).

### 2.1. Search Strategy

**Figure A1:**
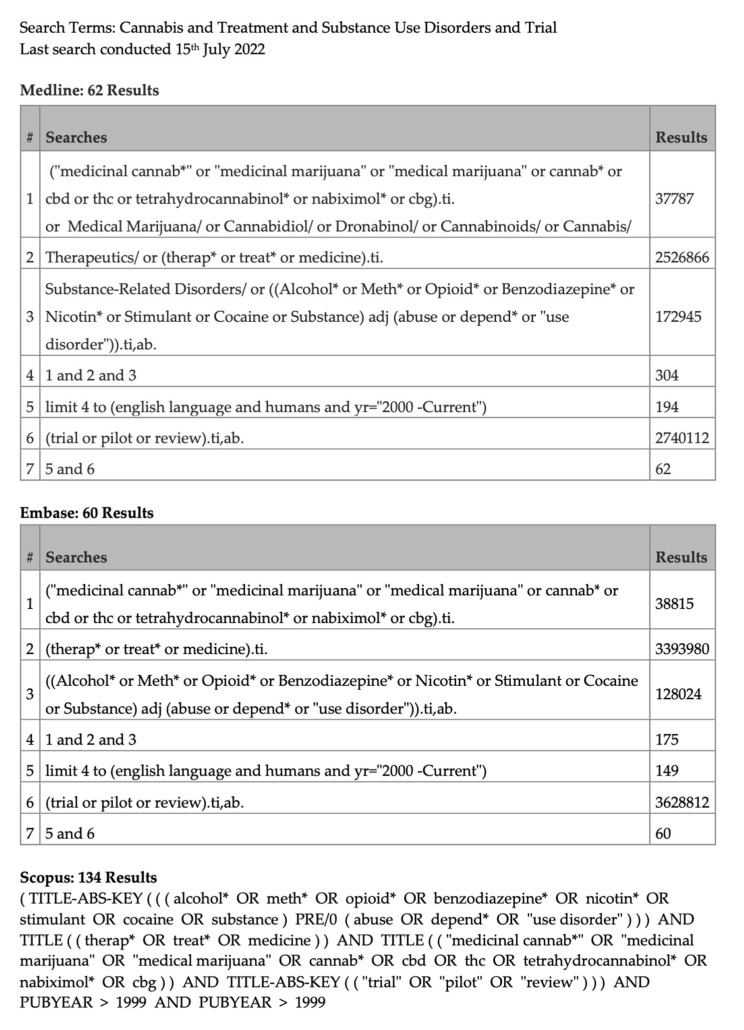
Search strategy.

For this systematic scoping review, we used a framework for systematic reviews and meta-analyses, the PRISMA guidelines, to inform our search methodology [18] and applied the Arksey and O’Malley [19,20,21] approach to performing a scoping review. In doing so we searched the Medline (Ovid), Embase (Ovid), and Scopus databases (Figure A1). An identical keyword search was applied to all three databases, with the added option of mesh terms leveraged in the Medline search. Studies published in English from January 2000 to July 2022 were considered for inclusion, as treatments that involved the use of cannabinoids for the treatment of substance-use disorders were not investigated prior to this time. Titles and abstracts of search results were screened independently by two researchers to determine whether they met the inclusion criteria. Accepted papers were retrieved, and further papers were excluded based on failure to meet inclusion criteria following full-text readings. Any additional papers referenced by these results meeting inclusion criteria were added to our search results.

The full-text articles that were identified for inclusion following the screening process were then independently critiqued by pairs of reviewers. A standardised format was used to extract and summarise their data according to PICOS [22]. Qualitative data synthesis was applied to each category of substance-use disorder. Due to the heterogeneity of outcome measures, no aggregated data analysis was performed.

### 2.2. Study Inclusion

Of the 243 articles identified in the search, 71 duplicate articles were removed using Endnote, and 128 were excluded following the screening of title and abstracts. This provided 44 potentially relevant full-text articles for screening prior to inclusion. Of these, 1 article was not able to be retrieved, and 21 were excluded as they did not meet criteria for study type, language, intervention intention, or were a non-clinical investigation (Table A1). After analysis of references across these 43 papers, an additional 1 SR and 2 NRs yielded were deemed as meeting inclusion criteria and incorporated into the search results, yielding a final total of 25 studies.

**Figure 1:**
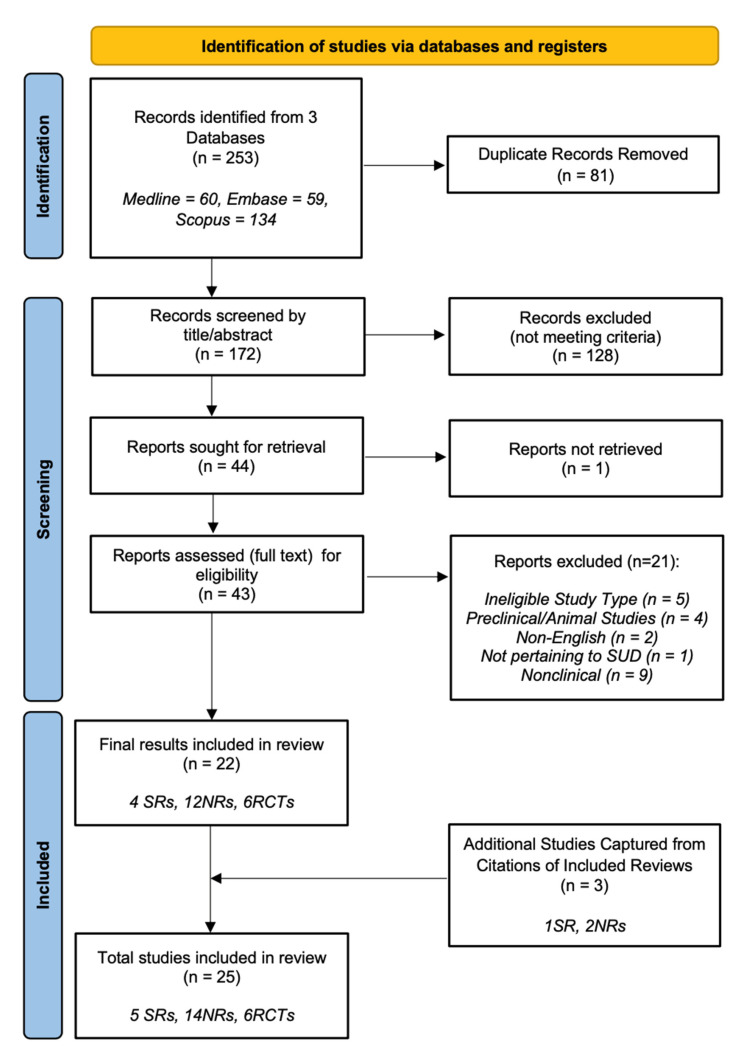
PRISMA flow chart of narrative review search methods.

The search strategy and results are presented in the PRISMA flow chart found in Figure 1.

Adapted from: Page MJ, McKenzie JE, Bossuyt PM, Boutron I, Hoffmann TC, Mulrow CD, et. al. The PRISMA 2020 statement: an updated guideline for reporting systematic reviews. BMJ 2021;372:n71. doi: 10.1136/bmj.n71. For more information, visit: http://www.prisma-statement.org/ (accessed on 24 August 2022).

## 3. Results

Of the 25 studies included in this review, 5 were systematic reviews, 14 were narrative reviews, and 6 were randomised controlled trials. The primary papers that were included in each of the systematic and narrative reviews captured by this study were evaluated and considered for inclusion in what is termed a ‘snowballing’ approach for identification of primary studies from systematic literature studies [23]. This yielded 23 further RCTs in addition to the 6 RCTs captured by the original search, yielding a total of 29 unique RCTs (Table 2).

### 3.1. Participants

The 29 unique RCTs included patients meeting criteria for a substance-use disorder (DSM-5), substance abuse, or dependence (DSM-IV) pertaining to the following substances: cannabis (13 RCTs), opioids (4 RCTs), cocaine (2 RCTs), nicotine (8 RCTs), and alcohol (2 RCTs).

### 3.2. Interventions

The 29 unique RCTs tested THC (3 RCTs), dronabinol (6 RCTs), nabiximols (5 RCTs), cannabidiol (7 RCTs), FAAH inhibitors (1 RCTs), rimonabant (5 RCT), surinabant (1 RCT), and taranabant (1 RCT). In 16 studies (55%) the endocannabinoid-based therapy was given in conjunction with a psychosocial intervention such as standard counselling and/or psychotherapy.

Table 3, Table 4, Table 5, Table 6 and Table 7 provide a summary of each paper that includes author names, title, date of publication, study type and study details including intervention type and duration, and outcome.

In this review, we categorised 29 research articles into five categories based on the type of substance-use disorder that was treated with medicinal cannabinoids. Due to the heterogeneity of outcome measures, no aggregated data analysis was performed.

### 3.3. Cannabis-Use Disorder

#### 3.3.1. Tetrahydrocannabinol (THC)

There were three small trials (n = 7,8,8) examining THC for cannabis-use disorder. These were short duration (<7 days), within-subject studies trialling THC for attenuation of cannabis withdrawal severity and cravings. Daily 50 mg doses of THC significantly decreased a number of withdrawal symptoms including anxiety, mood, chills, sleep disturbance, and anorexia [51]. A study comparing 30 mg/90 mg THC to placebo yielded similar results with a dose-dependent relationship, such that 90 mg reduced symptom ratings to that as if regular smoking practice was maintained [52]. A trial combining THC with lofexidine (an alpha-2 receptor agonist aiming to attenuate known noradrenergic hyperactivity in cannabinoid withdrawal) versus THC monotherapy reported similar effect in attenuating withdrawal symptoms, as well as significant reduction in relapse with the combination treatment but not with THC monotherapy [53]. All studies noted excellent tolerability with few side effects including cognitive effects.

#### 3.3.2. Dronabinol

Larger trials have examined dronabinol, a synthetic enantiomer of THC, for cannabis-use disorder. Two double-blinded RCTs in large populations (n = 156,122) of longer duration (9, 10 weeks) were conducted [54,56]. Both studies combined oral dronabinol (with additional lofexidine in the 2016 study) with Motivational Enhancement Therapy (MET) and Cognitive Behavioural Therapy (CBT) examining abstinence, cannabis use, and withdrawal severity. Although significant attenuation of withdrawal symptoms was observed, no significant difference in abstinence or cannabis use at two weeks was found versus placebo (although both groups showed reduction in cannabis use over the nine-week trial) [54]. No significant difference was found for combined dronabinol and lofexidine for withdrawal severity or abstinence [56].

#### 3.3.3. Cannabidiol

A single-phase 2a RCT has examined the effect of oral CBD for four weeks for cannabis-use disorder [57]. Higher doses (400, 800 mg compared with 200 mg daily) were shown to significantly reduce cannabis use as assessed by urinary THC-COOH: creatinine concentrations and reported days of cannabis use per week (−0.48 and −0.27 days, respectively).

#### 3.3.4. Nabiximols

Nabiximols were assessed in three small (n = 9,40,51) and one larger trial (n = 128). One study (n = 51) trialling 86.4 mg THC:80 mg CBD as a maximum daily dose for six days, showed withdrawal symptoms (measured by CWS (Cannabis Withdrawal Scale = 19 questions, each on a scale of 0–10)) significantly improved (mean 66% decrease from baseline levels) compared with placebo (mean 52% increase) [48]. However, no significant difference was found in cannabis use at follow-up 28 days later. The study with a fixed dose of 108 mg THC/100 mg CBD (n = 9) across eight weeks also reported attenuated withdrawal reducing the average CWS score to 10 from a baseline of 18 (with smoking as usual scoring 9). Effects on craving were not statistically significant [49]. The study of 40 patients given nabiximols (113.4 mg THC:105 mg CBD) also included MET/CBT for 12 weeks [50] but did not replicate findings described earlier. No significant differences in withdrawal scores or abstinence rates were found, but significant reduction in cannabis use across 12 weeks (−70.5% in nabiximols vs. −42.6% placebo) and cravings was found. The larger trial (n = 128) of 86.4 mg THC/80 mg CBD for 12 weeks also yielded non-significant findings for withdrawal symptoms, yet found significant reduction in craving and cannabis use across the trial (41.7% vs. 63.1% placebo days of usage across 12 weeks) [42]. A study following the same subjects three months after cessation of treatment found significant reduction in average number of days of cannabis use (−6.7 days in the previous 28 days) in addition to an increased proportion of subjects meeting criteria for abstinence (23% vs. 9% placebo) [43].

#### 3.3.5. Fatty-Acid Amide Hydrolase Inhibitor—PF-04457845

One single-phase 2a RCT (n = 46) treatment with 4 mg PF-04457845 oral capsules for four weeks reported it to be safe and well tolerated, with cannabis withdrawal severity and cannabis usage (both self-reported and urine verified) both significantly reduced (61.2% reduction in urinary THC-COOH concentration) [58].

### 3.4. Opioid-Use Disorder

#### 3.4.1. Dronabinol

Dronabinol (30 mg daily) for patients undergoing eight-day inpatient withdrawal and induction onto naltrexone continuing for five weeks post discharge, showed significant attenuation of the acute inpatient phase of withdrawal before naltrexone initiation (average SOWS (Subjective Opiate Withdrawal Scale = 16 questions, each on a scale of 0–4; mild withdrawal scores 1–10, moderate withdrawal scores 11–20, severe withdrawal scores 21–30) reduction of 11.34 vs. placebo) [44]. However, dronabinol did not improve symptoms of protracted low-grade withdrawal (insomnia, appetite, and low energy) that typically occur over the subsequent few weeks of outpatient treatment.

In a within-patient trial (n = 12) where a single dose of dronabinol (ranging from 5 to 40 mg, randomly assigned) was administered, subjects were assessed for physiological tolerability in one analysis [59] and withdrawal severity and psychomotor/cognitive effects in another [60]. Neither study found a difference compared to placebo for doses less than 20 mg. Larger doses did induce significant (albeit clinically moderate) withdrawal suppression; however, they also induced dose-dependent sustained symptomatic tachycardia and significant cognitive effects, specifically time estimation and continuous performance tasks.

#### 3.4.2. Cannabidiol (CBD)

An RCT (n = 42) assessed CBD for its potential impacts on craving, anxiety, cognition, affect, and physiological markers in abstinent opioid dependent individuals for a week following a three-day treatment with daily 400 mg or 800 mg CBD [61]. Significant reductions in craving (VAS-C (Visual Analog Scale for Craving = line 10 cm in length with ‘no craving’ and ‘severe craving’ at the extremes) mean difference 0.44 and 0.23, respectively), anxiety (VAS-A (Visual Analog Scale for Anxiety = line 10 cm in length with ‘not anxious’ and ‘very anxious’ at the extremes) mean difference 0.48 0.24, respectively) and physiological markers (heart rate, cortisol) were found, with no significant effect upon cognition or affect.

### 3.5. Cocaine-Use Disorder

#### Cannabidiol

A double-blinded study (n = 31) trialling CBD (300 mg daily) for crack-cocaine (crack- cocaine is a free-base form of cocaine that can be smoked, formulated by cooking cocaine powder with baking soda then breaking it into small pieces called ‘rocks’) dependence for 10 days reduced cue-induced craving severity but was not statistically significant [46]. Nor were there significant findings for anxiety, depression, or sleep disturbance. An RCT (n = 50) treating outpatients for 12 weeks with CBD (800 mg daily) also found no significant reduction in cue-induced craving severity or in time to relapse [47].

### 3.6. Nicotine-Use Disorder

#### 3.6.1. Rimonabant

Studies with Rimonabant and Tobacco Use (STRATUS) conducted multiple RCTs evaluating rimonabant for smoking cessation. STRATUS-EU and STRATUS-US were conducted with identical protocols and similar sample sizes (n = 784 and 783, respectively). Patients were treated with rimonabant (5 mg or 20 mg) daily for 10 weeks and followed up at 48 weeks for abstinence (both end of trial and prolonged), weight gain, and adverse events. STRATUS-META used only a 20 mg dose with no long-term follow-up after the 10-week intervention. A pooled analysis of these three trials showed 20 mg rimonabant significantly increased both end of treatment (OR = 1.6, 95% CI: 1.26, 2.12) and long-term (48 weeks) abstinence (OR = 1.50, 95% CI: 1.03, 2.17) [65]. Whilst a significantly greater likelihood of side effects including nausea (19.9% vs. 5.9% placebo) and anxiety (14.7% vs. 2.4% placebo) were found, no evidence of depressive symptoms was established. The STRATUS-WW (Worldwide) trial used a similar approach, demonstrating daily doses of 20 mg elicited significant improvement (RR for the 20 mg maintenance group was 1.29 (95% CI 1.06 to 1.57)) in the primary outcome of prevention of relapse to smoking.

#### 3.6.2. Taranabant

Another CB1 inverse agonist, taranabant, was trialled for eight weeks (2 vs. 4 vs. 8 mg daily) for dependent cigarette smokers and failed to demonstrate effect on end-of-trial abstinence [67]. This trial recorded significant neuropsychiatric (depression, irritability) and gastrointestinal (nausea, vomiting, diarrhoea) side effects.

#### 3.6.3. Surinabant

An RCT testing surinabant, a CB1 receptor antagonist and alternative to CB1 inverse agonists, used a similar protocol to the previously discussed taranabant trial and failed to have significant effect on abstinence over placebo [66]. Unlike the inverse agonists, Surinabant did not demonstrate significant neuropsychiatric side effects.

#### 3.6.4. Cannabidiol

The effect of CBD upon self-reported cigarette use in dependent cigarette smokers was recorded in a one-week study where participants were given access to ad hoc usage of a CBD inhaler [68]. A significant reduction in cigarette use (~40%) both at the end of the treatment week and at the two-week follow-up was demonstrated in the absence of significant effect on craving or mood related side effects (sedation, depression, anxiety). An RCT trialling a single dose of 800 mg in dependent cigarette smokers observed that whilst it had non-significant effects on craving or withdrawal, it significantly reduced both attentional bias towards and subjective pleasantness of cigarette-related stimuli [69].

### 3.7. Alcohol-Use Disorder

Rimonabant

A proof-of-concept study (n = 258) tested the efficacy of rimonabant (2 × 10 mg daily) in relapse prevention for alcohol dependence for 12 weeks. Rimonabant was deemed tolerable but had no significant effect upon relapse to first drink, relapse to heavy drinking, or drinking frequency [45]. A second study applying the same dose for two weeks amongst non-treatment-seeking alcohol-dependent individuals reported similar findings, namely a non-significant effect upon self-reported (via telephone) alcohol consumption [62].

## 4. Discussion

### 4.1. Cannabis-Use Disorder

#### 4.1.1. THC and Dronabinol

For cannabis-use disorder both THC and dronabinol (synthetic THC) appear to have benefit in attenuating withdrawal and cravings in a dose-dependent manner with good tolerability.

Neither cannabinoid, however, shows benefit in achieving abstinence from cannabis [29,30,31,32], which might have been expected given the success of opioid-substitution and nicotine-substitution treatments for their respective use disorders [5,6]. The dronabinol trials [54,56] cited cogent limitations accounting for their results. In the first, the authors postulated the short maintenance period (nine weeks) or the enrolment of non-treatment-seeking participants may have contributed to lack of abstinence. In the second, they suggested the failed result may have arisen because the intervention in this trial was introduced to induce abstinence, as opposed to prevent relapse in an already abstinent patient. Future studies of dronabinol for cannabis-use disorder could investigate an increased dose of dronabinol or, alternatively, other analogues of THC that have greater bioavailability and potency [56].

#### 4.1.2. Cannabidiol (CBD)

Significant reduction in cannabis use with CBD [57] is promising, with 200 mg found ineffective and a marginal superiority of 400 mg over 800 mg indicating an inverted-U dose–response curve. Treatment duration of longer than four weeks, however, requires investigation. The mechanism of action of CBD therapy has been postulated to be via reduction of the impact of drug-related cues in attentional bias and craving [61,70]. Alternative mechanisms may include the effect of CBD on modulation of other comorbid psychiatric symptoms in cannabis-use disorder such as anxiety.

#### 4.1.3. Nabiximols

Nabiximols show paradoxical findings. On one hand, Allsop et al., (2014) [48] and Trigo et al., (2016) [49] reported similar findings: a reduction in withdrawal severity but non-significant effects on long-term cannabis use. Conversely, Trigo et al., (2018) [50] and Lintzeris et al., (2019) [42] observed non-significant effects on withdrawal severity, yet a significant reduction in longer term cannabis use. Of note, both latter trials employed adjunctive psychotherapy in their intervention, and this may explain the longer-term improvements found. In the case of Trigo et al., (2018), the higher daily dosages (113.4 mg THC:105 mg CBD) may also have contributed to the beneficial effect on cannabis use, especially given the dose-dependent effect found. The high abstinence rate in the placebo group (>40%) indicates that the behavioural intervention was efficacious; therefore, future research should attempt to establish the role of nabiximols alone on abstinence. The question arises regarding why nabiximols had benefit in reducing cannabis use whilst fixed-dose dronabinol failed. Potential explanations include the flexible dose schedule, pharmacokinetic profile (higher bioavailability and more rapid onset of action), presence of additional whole-plant-extract components, and/or synergistic effects of THC with CBD [42].

#### 4.1.4. Fatty-Acid Amide Hydrolase Inhibitor—PF-04457845

D’Souza et al., (2019) [58] demonstrated that FAAH inhibitors reduced both withdrawal severity and cannabis use in humans with limited psychoactive effects, suggesting that this approach could be more effective than simple direct CB1R agonism. This concurs with evidence showing that possessing a genetic variation of FAAH with reduced enzyme expression/activity confers a significantly lower likelihood of developing cannabis-use disorder than in wild-type carriers [71]. It also aligns with preclinical research showing a beneficial effect of FAAH inhibitors upon withdrawal severity [72]. Future research should compare the two approaches within the context of a larger sample in an outpatient setting.

### 4.2. Opioid-Use Disorder

#### 4.2.1. Dronabinol

Dronabinol (30 mg) significantly reduced acute opioid withdrawal symptoms, but failed to have effect upon protracted low-grade withdrawal [44]. Potential explanations include that a threshold of symptom severity is necessary for dronabinol to be effective, a specificity of dronabinol for the symptoms of acute but not more chronic symptoms of opioid withdrawal, or participants developing tolerance to its effects after weeks of administration. Dose-dependent withdrawal suppression with dronabinol was found in other studies [59,60]; however, accompanying sustained tachycardia and anxiety/panic halted further investigation of dronabinol for opioid-use disorder.

#### 4.2.2. Cannabidiol

CBD attenuates heroin-seeking behaviour in response to drug-associated cues in animals with a history of heroin self-administration [73]. This is clinically relevant as environmental cues are one of the strongest precipitators of craving, contributing to relapse. CBD’s effect upon cue-induced cravings, safe pharmacological profile, and lack of hedonic properties is promising as a potential treatment for opioid-use disorder [61]. Future studies with larger sample sizes, objective opioid measures, in addition to subjective metrics and longer duration are needed to establish the efficacy of CBD in relapse prevention for opioid-use disorder.

### 4.3. Cocaine-Use Disorder

#### Cannabidiol

Multiple preclinical trials suggested the onset and maintenance of cocaine addiction are reinforced by the dopaminergic neuro-transmission system [74] and withdrawal is associated with impaired dopamine function [75,76]. Preclinical studies of CB1 receptors in the ventral tegmental area demonstrate that CB1 agonists stimulate dopaminergic neurons causing an increase in extracellular dopamine levels in the nucleus accumbens [77,78] and therefore could perhaps disrupt the dopaminergic mechanisms of cocaine addiction.

Human trials have not, however, supported these hypotheses. Two trials explored CBD for cocaine-use disorder in both inpatients [46] and outpatients [47]. Neither context, higher doses (800 mg vs. 300 mg) nor longer treatment period (12 weeks vs. 10 days), showed benefit. No significant reduction in outcomes of cue-induced craving severity and time to relapse was found. Potential explanations for lack of effect include dose (CBD has complex dose–response curves), frequency of administration (e.g., twice-daily administration may be more effective given CBD’s 3 h peak plasma concentration), or that the mechanism of action of CBD is not as effective in the context of stimulants as for other substance-use disorders [47].

### 4.4. Nicotine-Use Disorder

#### 4.4.1. Rimonabant

The STRATUS trials were promising for use of Rimonabant in smoking cessation. However, in June 2007 the FDA found significantly increased likelihood of suicidality for daily rimonabant (20 mg) taken for at least a year, leading Sanofi-Aventis to withdraw rimonabant [65]. Further trials evaluating other CB1 antagonists also ceased [79].

#### 4.4.2. Taranabant

Taranabant, being related to rimonabant, demonstrated neuropsychiatric effects of a similar nature to rimonabant [67]. This illustrates a CB1 inverse agonist class effect and is therefore not suitable as a pharmacotherapy for substance-use disorders.

#### 4.4.3. Surinabant

Surinabant, a CB1 receptor antagonist, showed no significant effect in nicotine-use disorder [66]. Preclinical trials demonstrated that while CB1 receptor activation is necessary for short-term induction of nicotine-incentive learning and reinforcement of drug-seeking behaviour in rats, other mechanisms become more significant for these behaviours after a few weeks [35]. Therefore, smokers who have been dependent for a matter of decades (as in this trial) perhaps lose sensitivity to endocannabinoid CB1 antagonism as an effective intervention.

#### 4.4.4. Cannabidiol

The reduction in cigarette use in outpatients using an ad hoc CBD inhaler for one week was significant [68]. Reduction in both attentional bias and subjective pleasantness of cigarette-related stimuli was also demonstrated [69]. This supports the postulate that CBD exerts anti-addictive effects by minimising the effect of drug cues (incentive salience model of drug addiction), in addition to preventing the indexing of the reinforcing value of a drug through pleasure. No effect upon withdrawal or craving was demonstrated, and a significant limitation was that only a single dose was provided. Further studies with a longer treatment period and a range of repeated doses are needed.

### 4.5. Alcohol-Use Disorder

#### Rimonabant

Animal models show rimonabant decreases sensitivity to appetitive reinforcers [80] and reduces voluntary ethanol consumption [81,82]. Human trials have not, however, supported these findings [45,62]. A potential explanation for its limited effectiveness is that the higher doses used in animal studies enable near-complete receptor occupancy [62], yet higher doses of rimonabant in humans cannot be used because of the well documented adverse psychological effects.

### 4.6. Limitations

This review was limited by the vast heterogeneity across all primary studies included in this review. The populations varied in their specific diagnoses, treatment motivation, abstinence status, and degree of concurrent substance use. With respect to the intervention, there was significant variation in doses, adjunct interventions utilized, treatment setting, and duration. With respect to outcomes, differences included the specific outcomes assessed, how they were defined, follow-up duration, and the metric used to measure the outcomes. Most published reviews were inconclusive due to this heterogeneity, and quantitative data aggregation could not be undertaken.

### 4.7. Future Research Directions

The volume of research into endocannabinoids for substance-use disorders has failed to match the rapidly evolving public interest into their applications. Multiple states in the U.S. have decriminalized recreational use, and from a medical perspective the FDA has already approved multiple endocannabinoid formulations including Epidiolex (a CBD formulation) and Marinol (a dronabinol formulation). Given this rising tide and changing public opinion, the volume of primary research into the applications of endocannabinoids for substance-use disorders is set to increase. Such research will need to address the limitations in current research specified in 4.6 —this includes rigorous definitions of study populations, exploration of a range of dosages and adjunct interventions, and the application of standardised metrics for outcomes such as abstinence, withdrawal symptoms, and side effects.

### 4.8. Implications for Clinical Practice

The primary research thus far hints at the future potential for multiple endocannabinoid formulations across a range of substance-use disorders. Whilst there are many promising results, the paucity of human studies means that optimal dosages and treatment protocols are still yet to be researched and established. This is clinically critical not only from the perspective of optimising efficacy, but also in ensuring that side effects are either identified and characterised or alternatively deemed irreconcilable as in the case of rimonabant [79]. Therefore, given this absence in both volume and nuance of research it can be asserted that it is not yet appropriate to prescribe endocannabinoids as a clinician treating substance-use disorders. However, as future research moves to address these deficiencies and establish empirical validity, endocannabinoids will likely soon become a tool that is available to clinicians for both treating withdrawal and bolstering abstinence maintenance in the context of multiple-substance-use disorders. Therefore, it is worthwhile for such clinicians to vigilantly monitor the developing literature. Beyond efficacy, clinicians will next have to consider the socio-political dimension of their prescriptions including societal taboos and the interplay with the legal system (e.g., driving laws), and must be prepared to conduct evidence-based discussions with patients undoubtedly possessing a variety of preconceptions [83].

## 5. Conclusions

The body of evidence from human trials targeting the endocannabinoid system to treat substance-use disorders is not large and currently shows mixed results. The most promising research exists for cannabis-use disorder, indicating benefits of CB1R agonist therapy (dronabinol) for cannabis withdrawal. Potential application of nabiximols (whole-plant extract) and cannabidiol for reduction of cannabis use also exists. For opioids, direct CB1 agonist therapy at effective doses appears to induce intolerable side effects; however, CBD may have potential for reducing opioid cue-induced craving. Rimonabant was the only therapy that had been trialled in the context of alcohol-use disorder, but is not feasible because of significant psychotropic effects, hence its withdrawal from the market. The same applies for nicotine dependence. There is little promise thus far for the use of cannabinoids in cocaine-use disorder. Most of the examined studies, however, possessed small samples and multiple limitations; hence further studies of medicinal cannabinoids as a treatment option for substance-use disorders are needed moving into the future.

## Figures and Tables

**Table 1:** PICOS Search algorithm–scoping review using a systematic approach.

Search Parameter	Inclusion Criterion
Participants	Studies were included if their participants met the criteria for a substance-use disorder (DSM-5), abuse, or dependence (DSM-IV).
Interventions	Experimental condition: any formulation with primary mechanism of action upon the endocannabinoid system (receptors, ligands, or enzymes) with the intention to treat a substance-use disorder, abuse, or dependence.
Control	Placebo or any other intervention differing from the experimental condition.
Outcome	The primary outcome was the effectiveness of the intervention for the treatment of any substance-use disorder.
Study design	The studies considered for inclusion in this review were systematic reviews (SRs), narrative reviews (NRs), and randomised-control trials (RCTs)

**Table 2:** Primary studies included in scoping review.

Primary Papers N = 29	Systematic Scoping Review Search Results N = 25																								
	Systematic Reviews n = 5					Narrative Reviews n = 14														Randomised Controlled Trials n = 6					
	Batalla et. al. 2019, [24]	Hoch et. al., 2019 [25]	Paulus et. al., 2022 [26]	Pavel et. al., 2021 [27]	Prud’hommeet. al., 2015 [28]	Babalonis & Walsh 2020 [29]	Beardsley et. al., 2009 [30]	Calpe-López et al., 2019 [31]	Chye et. al., 2019 [32]	Femenia et. al., 2009 [33]	Fischer et. al., 2015 [34]	Foll et. al., 2008 [35]	Galaj & Xi 2019 [36]	Kleczko-ska et. al., 2015 [37]	Kolongo-wski et. al., 2021 [38]	Lee et. al., 2017 [39]	Navarrete et. al., 2021 [40]	Sholler et. al., 2020 [41]	Sloan et. al., 2017 [9]	Lintzeris et. al., 2019 [42]	Lintzeris et. al., 2020 [43]	Bisaga et. al., 2015 [44]	Soyka et. al., 2008 [45]	Meneses-Gaya et. al., 2021 [46]	Mongeau-Pérusse et. al., 2021 [47]
Allsop et. al. 2014 [48]																									
Trigo et. al., 2016 [49]																									
Trigo et. al., 2018 [50]																									
Lintzeris et. al., 2019 [42]																									
Lintzeris et. al., 2020 [43]																									
Haney et. al., 2003 [51]																									
Budney et. al., 2007 [52]																									
Haney et. al., 2007 [53]																									
Levin et. al., 2011 [54]																									
Vandrey et. al., 2013 [55]																									
Levin et. al., 2016 [56]																									
Freeman et. al., 2020 [57]																									
D’Souza et. al., 2019 [58]																									
Bisaga et. al., 2015 [44]																									
Jicha et. al., 2015 [59]																									
Lofwall et. al., 2016 [60]																									
Hurd et. al., 2019 [61]																									
Soyka et. al., 2008 [45]																									
George et. al., 2009 [62]																									
Meneses-Gaya et. al., 2021 [46]																									
Mongeau-Pérusse et. al., 2021 [47]																									
Rigotti et. al., 2009 [63]																									
Cahill & Ussher 2011 [64]																									
Robinson et. al., 2017 [65]																									
Tonstad & Aubin 2012 [66]																									
Morrison et. al., 2010 [67]																									
Morgan et. al., 2013 [68]																									
Hindocha et. al., 2018a [69]																									
Hindocha et. al., 2018b [70]																									

Legend: Substance of use disorder—green = cannabis (13 RCTs); blue = opioids (4 RCTs); orange = alcohol (2 RCTs); red = cocaine (2 RCTs); purple = nicotine (8 RCTs).

**Table 3:** Primary Studies pertaining to Cannabis Use Disorder.

Study	Type	N	Population	Intervention	Adjunct Intervention	Duration	Follow Up	Comparator	Outcomes	Findings
Nabiximols										
Allsop et. al., 2014 [48]	Randomised Control Trial (RCT)	51	Cannabis Dependence (DSM-IV), Treatment Seeking	Nabixi-mols, oro-mucosal spray, 86.4 mg tetrahydrocannabinol (THC):80 mg can-nabidiol (CBD) (max dai-ly dose)	CBT Workbook, Standard Detoxification Care	6 days	28 days	Placebo	*Primary:* Withdrawal Severity (CWS) *Secondary*: Cannabis Use (28 day follow-up) Treatment Retention	↓ Withdrawal Severity =Cannabis Use ↑ Treatment Retention
Trigo et. al., 2016 [49]	RCT	9	Cannabis Dependence (DSM-IV), Non-Treatment Seeking	Nabiximols, Oromucosal Spray, 100 mg CBD:108 mg THC (max daily dose)	n/a	8 weeks	n.a.	Placebo	*Primary:* Withdrawal Severity (CWS) *Secondary:* Craving (MCQ)	↓ Withdrawal Severity (dose-dependent) =Craving (MCQ)
Trigo et. al., 2018 [50]	RCT	40	Cannabis Dependence (DSM-IV), Treatment Seeking	Nabiximols Oromucusal Spray, 113.4 mg THC:105 mg CBD (max daily dose)	MET CBT	12 weeks	n.a.	Placebo	*Primary* Tolerability Abstinence (EOT) *Secondary* Cannabis Use (days/week) Withdrawal Severity (CWS) Craving (MCQ)	=Tolerability =Abstinence ↓ Cannabis Use = Withdrawal Severity ↓ Craving
Lintzeris et. al., 2019 [42]	RCT	128	Cannabis Dependence (DSM-IV), Treatment Seeking	Nabiximols, Oromucosal Spray, 80 mg CBD:86.4 mg THC (maximum daily doses),	CBT, Case Management	12 weeks	n.a.	Placebo	*Primary:* Cannabis Use (days/trial) *Secondary:* Craving (MCQ) Withdrawal Severity (CWS)	↓ Cannabis Use = Craving = Withdrawal Severity
Lintzeris et. al., 2020 [43]	RCT	128	Cannabis Dependence (DSM-IV), Treatment Seeking	Nabiximols, Oromucosal Spray, 80 mg CBD:86.4 mg THC (maximum daily doses),	CBT, Case Management	12 weeks	24weeks	Placebo	*Primary:* Cannabis Use *Secondary:* Abstinence (previous 28 days)	↓ Cannabis Use ↑ Abstinence
Tetrahydrocannabinol (THC)										
Haney et. al., 2003 [51]	Placebo Controlled, Within Subject Study	7	Cannabis Users, Non-Treatment Seeking	THC, Oral Capsules, 5 × 10 mg	n/a	6 days	n.a.	Placebo	*Primary:* Withdrawal Severity (Marijuana Withdrawal Checklist) *Secondary:* Craving	↓ Withdrawal Severity ↓ Craving
Budney et. al., 2007 [52]	Placebo Controlled, Within Subject Study	8	Cannabis Dependence (DSM-IV), Non-Treatment Seeking	THC, Oral Capsules, 30 mg vs. 90 mg	n/a	5 days	n.a.	Placebo	*Primary:* Withdrawal Severity (Marijuana Withdrawal Checklist) *Secondary:* Craving (Marijuana Craving Questionnaire)	↓ Withdrawal Severity (dose-dependent) ↓ Craving
Haney et. al., 2007 [53]	Placebo Controlled, Within Subject Study	8	Cannabis Users, Non-Treatment Seeking	THC 3x20 mg vs Lofexidine 2.4 mg vs THC + Lofexidine	n/a	7 days	n.a.	Placebo	*Primary:* Withdrawal Severity (Marijuana Withdrawal Checklist) *Secondary:* Relapse Cannabis Use Craving (VAS)	↓ Withdrawal Severity (all combinations) ↓ Relapse Cannabis Use (Lofexidine, THC + Lofexidine) ↓ Craving (Lofexidine, THC + Lofexidine)
Dronabinol										
Levin et. al., 2011 [54]	RCT	156	Cannabis Dependence (DSM-IV), Treatment Seeking	Dronabinol, Oral Capsules, 2 × 20 mg	MET, Relapse Prevention Therapy	9 weeks	n.a.	Placebo	*Primary:* Abstinence (2 weeks, EOT) *Secondary:* Cannabis Use (Self-Reported) Withdrawal Severity (Withdrawal Discomfort Score)	=Abstinence =Cannabis Use ↓ Withdrawal Severity
Vandrey et. al., 2013 [55]	Placebo Controlled, Within Subject Study	13	Cannabis Dependence (DSM-IV), Non-Treatment Seeking	Dronabinol, Oral Capsules, 30 vs. 60 vs. 120 mg	n/a	5 days	n.a.	Placebo	*Primary:* Withdrawal Severity (Marijuana Withdrawal Checklist)	↓ Withdrawal Severity (dose-dependent)
Levin et. al., 2016 [56]	RCT	122	Cannabis Dependence (DSM-IV), Treatment Seeking	Dronabinol(3 × 20 mg) +Lofexidine(3 × 0.6 mg)	MET, Relapse Prevention Therapy	10 weeks	n.a.	Placebo	*Primary:* Abstinence (3 weeks, EOT) *Secondary*: Withdrawal Severity	=Abstinence = Withdrawal Severity
Cannabidiol (CBD)										
Freeman et. al., 2020 [57]	Phase 2a, double-blind, placebo-controlled, randomized, adaptive Bayesian trial	48	CUD (DSM-V),Treatment Seeking	CBD, Oral Capsules, 200 vs. 400 vs. 800 mg	Motivational Interviewing	4 weeks	n.a.	Placebo	*Primary:* Cannabis Use (urinary THC-COOH: creatinine conc) *Secondary:* Withdrawal Severity (Cannabis Withdrawal Scale)	↓ Cannabis Use (400 mg, 800 mg) ↓ Withdrawal Severity (800 mg)
Fatty Acid Amide Hydrolase (FAAH) Inhibitor										
D’Souza et. al., 2019 [58]	Phase 2a, double-blind, placebo-controlled, randomized trial	46	Cannabis Dependence (DSM-IV), Treatment Seeking	PF-04457845,Oral Capsules, 4 mg	n/a	4 weeks	n.a.	Placebo	*Primary:* Cannabis Withdrawal Severity *Secondary:* Cannabis Use (Urine + Self-Reported)	↓ Cannabis Withdrawal ↓ Cannabis Use

**Table 4:** Primary Studies pertaining to Opioid Use Disorder.

Study	Type	N	Population	Intervention	Adjunct Intervention	Duration	Follow Up	Comparator	Outcomes	Findings
Dronabinol										
Bisaga et. al., 2015 [44]	RCT	60	Opioid Dependence (DSM-IV), Treatment Seeking	Dronabinol, Oral Capsule30 mg	MET, CBT, Relapse Prevention Therapy	8 weeks.	3 weeks.	Placebo	*Primary* Withdrawal Severity (SOWS) Naltrexone Treatment Retention	↓ Withdrawal Severity = Naltrexone Treatment Retention
Jicha et. al., 2015 [59]	Within Subject RCT	12	Opioid Dependence (DSM-IV), Non-Treatment Seeking	Dronabinol, Oral Capsule5 vs. 10 vs. 20 vs. 30 mg (40 mg discontinued)	n/a	Single Dose	n.a.	Placebo, Oxycodone 30 vs. 60 mg	Physiological Tolerability	↑ Heart Rate (>=20 mg) = Physiological Parameters (<20 mg)
Lofwall et. al., 2016 [60]	Within Subject RCT	12	Opioid Dependence (DSM-IV), Non-Treatment Seeking	Dronabinol, Oral Capsule5 vs. 10 vs. 20 vs. 30 mg (40 mg discontinued)	n/a	Single Dose	n.a.	Placebo, Oxycodone 30 vs. 60 mg	Withdrawal Severity (SOWS) Psychomotor/Cognitive Effects	↓Withdrawal Severity (>=20 mg) ↑Psychomotor/Cognitive Effects
Cannabidiol (CBD)										
Hurd et. al., 2019 [61]	RCT	42	Opioid Dependence (DSM-IV)	Cannabidiol, Oral Solution, 400,800 mg	n/a	3 days	7 days	Placebo	Primary Cue Induced Craving (HCQ) Anxiety (VAS-A) Secondary Cognition Affect Physiological Markers (Heart Rate, Cortisol)	↓ Cue Induced Craving3 ↓ Anxiety =Cognition =Affect ↓Physiological Markers (Heart Rate, Cortisol)

**Table 5:** Primary Studies pertaining to Alcohol Use Disorder.

Study	Type	N	Population	Intervention	Adjunct Intervention	Duration	Follow Up	Comparator	Outcomes	Findings
Rimonabant										
Soyka et. al., 2008 [45]	Phase 2a RCT	258	Alcohol Dependence (DSM-IV), Recently Detoxified	Rimonabant, Oral Capsule, 2 × 10 mg	n/a	12 weeks	n.a.	Placebo	*Primary* Relapse to First Drink Relapse to Heavy Drinking *Secondary* Alcohol Consumption	=Relapse to First Drink =Relapse to Heavy Drinking =Alcohol Consumption
George et. al., 2009 [62]	Phase I/II RCT	49	Alcohol Dependence/Abuse (DSM-IV), Non-Treatment Seeking	Rimonabant, Oral Capsule, 20 mg	n/a	2 weeks	n.a.	Placebo	*Primary* Alcohol Consumption	=Alcohol Consumption

**Table 6:** Primary Studies pertaining to Cocaine Use Disorder.

Study	Type	N	Population	Intervention	Adjunct Intervention	Duration	Follow Up	Comparator	Outcomes	Findings
Cannabidiol (CBD)										
Meneses-Gaya et. al., 2021 [46]	RCT	31	Crack-Cocaine Dependence (DSM-IV)	CBD, Oral Solution, 300 mg	n/a	10 days	n.a.	Placebo	*Primary* Cue Induced Craving Severity	=Cue Induced Craving Severity
Mongeau-Pérusse et. al., 2021 [47]	Phase II RCT	50	Cocaine Use Disorder (DSM-V	CBD, Oral Solution, 800 mg	Group Therapy	12 weeks	n.a.	Placebo	*Primary* Cue Induced Craving Severity *Secondary* Time to Relapse	=Cue Induced Craving Severity =Time to Relapse

**Table 7:** Primary Studies pertaining to Nicotine Use Disorder.

Study	Type	N	Population	Intervention	Adjunct Intervention	Duration	Follow Up	Comparator	Outcomes	Findings
Rimonabant										
Rigotti et. al., 2009 [63]	RCT	755	Nicotine Dependence (DSM-IV), Treatment Seeking	Rimonabant 20 mg + Nicotine Patch	Smoking Counselling	10 weeks	13 weeks	Rimonabant 20 mg + Placebo Patch	*Primary* Abstinence (EOT, 4 Week Continuous) *Secondary* Point Prevalence Abstinence (weeks 9,24) Sustained Abstinence (weeks 6-24) Weight Change	↑ Abstinence (all measures) =Weight Change
STRATUS-WW 2005 [64]	Double-blind placebo-controlled parallel-assignment RCT	5055	Smokers (>10cpd), Treatment Seeking	Rimonabant5 vs. 20 mg	Behavioural Counselling	Phase 1: 10 weeks Phase 2: 42 weeks	104 weeks	Placebo	*Primary* Relapse Prevention Rate *Secondary* Weight Change	↑ Relapse Prevention Rate (20 mg) ↓ Weight Gain (20 mg)
STRATUS-EU 2006 [64,65]	Double-blind placebo-controlled parallel-assignment RCT	783	Smokers (>10cpd), Treatment Seeking	Rimonabant5 vs. 20 mg	Behavioural Counselling	10 weeks	48 weeks	Placebo	*Primary* Abstinence at EOT (10 weeks) and Prolonged (48 weeks) *Secondary* Weight Gain Adverse Events (GI Disturbance, Anxiety)	↑ Abstinence (EOT & Prolonged) (20 mg) ↓ Weight Gain (20 mg) ↑ Adverse Events (20 mg)
STRATUS-US 2006 [64,65]	Double-blind placebo-controlled parallel-assignment RCT	784	Smokers (>10cpd), Treatment Seeking	Rimonabant5 vs. 20 mg	Behavioural Counselling	10 weeks	48 weeks	Placebo	*Primary* Abstinence at EOT (10 weeks) and Prolonged (48 weeks) *Secondary* Weight Gain Adverse Events (GI Disturbance, Anxiety)	↑ Abstinence (EOT & Prolonged) (20 mg) ↓ Weight Gain (20 mg) ↑ Adverse Events (20 mg)
STRATUS-META 2006 [65]	Double-blind placebo-controlled parallel-assignment RCT	530	Smokers (>10cpd), Treatment Seeking	Rimonabant20mg	Behavioural Counselling	10 weeks	n.a.	Placebo	*Primary* Abstinence at EOT (10 weeks) *Secondary* Weight Gain Adverse Events (GI Disturbance, Anxiety)	↑ Abstinence (EOT) ↓ Weight Gain ↑ Adverse Events
Surinabant										
Tonstad & Aubin, 2012 [66]	Double-blind placebo-controlled parallel-assignment RCT	810	Smokers (>10cpd	Surinabant 2.5 vs. 5 vs. 10 mg	Smoking Cessation Counselling	8 weeks	6 weeks	Placebo	*Primary* Abstinence (EOT, 4 weeks continuous) *Secondary* Weight Gain Neuropsychiatric SE	=Abstinence ↓ Weight Gain =Neuropsychiatric SE
Taranabant										
Morrison et. al., 2010 [67]	RCT	317	Dependent Cigarette Smokers	Taranabant, Oral Capsules, 2 vs. 4 vs. 8 mg + Counselling	Smoking Cessation Counselling	8 weeks	6 weeks	Placebo	*Primary* Abstinence (EOT, 4 weeks continuous) *Secondary* Weight Gain Neuropsychiatric SE (Depression) Gastrointestinal SE	=Abstinence ↓ Weight Gain ↑ Neuropsychiatric SE ↑Gastrointestinal SE
Cannabidiol (CBD)										
Morgan et. al., 2013 [68]	RCT	24	Dependent Cigarette Smokers, Non-Treatment Seeking	CBD, Inhaler, Ad Hoc Use	Smoking Cessation Counselling	1 week	2 weeks	Placebo	*Primary* Cigarette Usage *Secondary* Craving Mood Side Effects (Sedation, Depression, Anxiety)	↓ Cigarette Usage =Craving =Mood Side Effects
Hindocha et. al., 2018a [69]	RCT Double Blind Cross-Over Design	30	Dependent Cigarette Smokers, Non Treatment Seeking	CBD, 800 mg	n/a	Single Dose	n.a.	Placebo	*Primary* Attentional Bias to Cigarette Cues during Abstinence Pleasantness of Cigarette Stimuli during Abstinence Craving Withdrawal Side Effects	↓ Attentional Bias to Cigarette Cues during Abstinence ↓Pleasantness of Cigarette Stimuli during Abstinence =Craving =Withdrawal =Side Effects
Hindocha et. al., 2018b [70]	RCT Double Blind Cross-Over Design	30	Dependent Cigarette Smokers, Non Treatment Seeking	CBD, 800 mg	n/a	Single Dose	n.a.	Placebo	Verbal and Spatial Working Memory Impulsivity	=Verbal and Spatial Working Memory =Impulsivity

## Data Availability

All relevant data within the manuscript are available on request.

## Appendix A

**Table A1:** Excluded studies.

NO.	Author	Reason for exclusion
1	Bhardwaj et. al., 2018 [84]	Ineligible study type—study protocol.
2	Calpe-López et. al., 2019 [31]	Nonclinical—pharmacological review of mechanisms.
3	Calpe-López et. al., 2019 [31]	Duplicate.
4	Cohen et. al., 2020 [85]	Not pertaining to SUD treatment, only PTSD.
5	De Ternay et. al., 2019 [86]	Animal studies—no human trials.
6	George 2007 [87]	Ineligible study type—book chapter.
7	Janero 2012 [88]	Ineligible study type—short survey.
8	Janero & Makriyannis, 2007 [89]	Nonclinical—pharmacological review of mechanisms.
9	Khurana et. al., 2017 [90]	Nonclinical—pharmacological review of mechanisms.
10	Lake et. al., 2021 [91]	Ineligible study type—cohort study.
11	Lee et. al., 2017 [39]	Nonclinical—pharmacological review of mechanisms.
12	Luján & Valverde, 2020 [92]	Nonclinical—pharmacological review of mechanisms.
13	Mackie 2006 [93]	Nonclinical—pharmacological review of mechanisms.
14	Onaivi 2009 [94]	Nonclinical—pharmacological review of mechanisms.
15	Pietrzak et. al., 2011 [95]	Non-English (Polish).
16	Preedy 2017 [96]	Ineligible study type—book chapter.
17	Rodrigues et. al., 2020 [97]	Animal studies—no human trials.
18	Sholler et. al., 2020 [41]	Nonclinical—pharmacological review of mechanisms.
19	Śmiarowska et. al., 2022 [98]	Nonclinical—pharmacological review of mechanisms.
20	Weidenauer et. al., 2021 [99]	Non-English (German).
21	Yang et. al., 2012 [100]	Animal studies—no human trials.
